# Supplementary material for: Safety and Efficacy of SGLT2 Inhibitors: A Multiple-Treatment Meta-Analysis of Clinical Decision Indicators
Source: J Clin Med. 2021 Jun 19;10(12):2713. doi: 10.3390/jcm10122713 (PMC8233997; doi:10.3390/jcm10122713)
Supplement: Supplementary file 1 [file jcm-10-02713-s001.zip › jcm-1233150-supplementary.pdf]

Supplementary material

Table S1. Search strategy for the MEDLINE database

Search Set Medline	
#1	T2D [All Fields]
#2	Type 2 diabetes [All Fields]
#3	Type 2 diabetes mellitus [All Fields]
#4	Diabetes mellitus type 2 [All Fields]
#5	1 OR 2 OR 3 OR 4
#6	RCT [All Fields]
#7	Randomized controlled trial [All Fields]
#8	6 OR 7
#9	Sodium-Glucose Transporter 2 [All Fields]
#10	Sodium-glucose cotransporter 2 inhibitor [All Fields]
#11	SGLT-2 inhibitors [All Fields]
#12	SGLT2 inhibitors [All Fields]
#13	Gliflozin [All Fields]
#14	Dapagliflozin [All Fields]
#15	Canagliflozin [All Fields]
#16	Empagliflozin [All Fields]
#17	Ipragliflozin [All Fields]
#18	Remogliflozin [All Fields]
#19	Tofogliflozin [All Fields]
#20	Sergliflozin [All Fields]
#21	Ertugliflozin [All Fields]
#22	Luseogliflozin [All Fields]
#23	Bexagliflozin [All Fields]
#24	9 OR 10 OR 11 OR 12 OR 13 OR 14 OR 15 OR 16 OR 17 OR 18 OR 19 OR 20 OR 21 OR 22 OR 23
#25	MACE [All Fields]
#26	Major adverse cardiovascular events [All Fields]
#27	Major adverse cardiac events [All Fields]
#28	25 OR 26 OR 27
#29	Mortality [Mesh Terms]
#30	Mortality rate [All Fields]
#31	Mortalities [All Fields]
#32	29 OR 30 OR 31
#33	5 AND 8 AND 24 AND 28 AND 32

Table S2. Characteristics of the included studies.

	EMPA-REG OUTCOME	CANVAS and CANVAS-R	CREDENCE	DECLARE-TIMI 58	VERTIS-CV
**Drug**	Empagliflozin	Canagliflozin	Canagliflozin	Dapagliflozin	Ertugliflozin
**n of participants in intervention by doses / n placebo**	10 mg: 2345 / 25 mg: 2342 / Placebo: 2333	100 mg or 300 mg: 5795 / Placebo: 4347	100 mg: 2202 / Placebo: 2199	10 mg: 8582 / Placebo: 8578	5 mg: 2742 / 15 mg: 2747 / Placebo: 2747
**Sponsor**	Boehringer Ingelheim	Janssen Research & Development, LLC	Janssen Research & Development, LLC	AstraZeneca	Merck Sharp & Dohme Corp.
**Type of patients**	T2DM patients (≥ 18 years of age) with established CVD.	T2DM patients (≥ 30 years of age) with established CVD or ≥ 50 years with ≥ 2 CVD.	T2DM patients (≥ 30 years of age) with CKD and albuminuria.	T2DM patients (≥ 40 years of age) with established CVD or multiple risk factors for CVD.	T2DM patients (≥ 40 years of age) with established atherosclerotic CVD.
**Median follow-up, years**	3.1	2.4	2.6	4.2	3.0
**Baseline age, mean ± SD**	63.1±8.6	63.3±8.3	63.0±9.2	63.9±6.8	64.4±8.1
**Baseline eGFR (ml/min/1.73 m^2)**	74±21	76.5±20.5	56.2 ± 18.2	85.2±15.9	76.0±20.9
**Baseline HbA1c mmol/mol (%)**	64.7 (8.07± 0.85)	66.1 (8.2±0.9)	67.2 (8.3 ±1.3)	67.2 (8.3±1.2)	67.2 (8.3±0.9)
**Patients with established CVD (%)**	>99%	65.6%	50.4%	40.6%	99.9%
**All-cause mortality**	28.6 / 19.5 rate/1000p-y	19.5 / 17.3 rate/1000 p-y	35.0 / 29.0 events/1000 p-y	16.4 / 15.1 rate/ 1000 p-y	26 / 24 events/1000 p-y
**MACE[1] placebo vs. treatment**	43.9 / 37.5 rate/1000p-y	31.5 / 26.9 rate/1000 p-y	48.7 / 38.7 events/1000 p-y	24.2 / 22.6 rate/ 1000 p-y	40 / 39 events/1000 p-y
**Hospitalization for heart failure**	14.5 / 9.4 rate/1000p-y	8.7 / 5.5 rate/1000 p-y	25.3 / 15.7 events/1000 p-y	8.5 / 6.2 rate/ 1000 p-y	11 / 7.5 events/1000 p-y
**Composite Renal outcome**					
**Definition**	Doubling of serum creatinine level, eGFR of ≤45, RRT, or death from renal disease.	40% reduction in eGFR, RRT, or renal death.	Doubling of the serum creatinine level, ESRD, or death from renal or cardiovascular disease.	≥40% decrease in eGFR to <60 ml/min/1.73 m2, ESRD, or death from renal cause.	Doubling of the serum creatinine level, RRT or death from renal causes.
**Placebo vs. treatment**	11.5 / 6.3 rate/1000 p-y	9.0 / 5.5 rate/1000 p-y	61.2 / 43.2 events/1000 p-y	7.0 / 3.7 rate/1000 p-y	12 / 9.5 events/1000 p-y
**Serious AE treatment/placebo (%)**	38.2% / 42.3%	24.6% / 26.7%	33.5% / 36.7%	34.1% / 36.2%	34.5% / 36.1%
**Acceptability**					
**Participants**	7020	10142	4401	17160	8246
**Completed alive**	6514 (92.8%)	9387 (92.6%)	3992 (90.7%)	15807 (92.1%)	7212 (87.4%)
**Deaths**	295 (4.2%)	347 (3.4%)	369 (8.4%)	1099 (6.4%)	727 (8.8%)
**Non completed[2]**	211 (3.0%)	408 (4.0%)	40 (0.9%)	254 (1.5%)	302 (3.7%)

Abbreviations: CVD: cardiovascular disease; CKD: chronic kidney disease; eGFR: estimated glomerular filtration rate (mL/min/1.73 m^2); CVD: cardiovascular disease; AE: Adverse event; p-t: patient-year; MACE: major adverse cardiovascular event; RRT: renal-replacement therapy; ESRD: end-stage renal disease (dialysis ≥30 or 90 days or kidney transplantation or confirmed sustained eGFR <15 mL/min/1.73 m^2).

[1] Defined as death from cardiovascular causes, nonfatal myocardial infarction, or nonfatal stroke.

[2]Deaths are not included.

Table S3. Quality assessment of the included studies assessed using the Cochrane Collaboration's tool.

	Random sequence generation	Allocation concealment	Blinding participants	Blinding outcome	Incomplete outcome data	Selective reporting	Other sources
**Zinman B et al. 2015**	Low	Low	Low	Low	Low	Low	Low
**Wanner C et al. 2016**	Low	Low	Low	Low	Low	Low	Low
**Neal B et al. 2017**	Low	Low	Low	Low	Low	Low	Low
**Perkovic V et al. 2017**	Low	Low	Low	Low	Low	Low	Low
**Wiviott SD et al. 2019**	Low	Low	Low	Low	Low	Low	Low
**Cannon CP et al. 2020**	Low	Low	Low	Low	Low	Low	Low

Table S4. Pooled hazard ratio of main outcomes. In upper right triangle, pooled hazard ratios from pairwise comparisons (column intervention relative to row); in lower left triangle pooled hazard ratios from the network meta-analysis (row intervention relative to column).

A. All-cause mortality

Pooled	**0.85 (0.75, 0.97)**			
Placebo	0.87 (0.74, 1.01)	0.93 (0.82, 1.04)	**0.68 (0.57, 0.82)**	0.93 (0.80, 1.08)
0.88 (0.61, 1.28)	Canagliflozin	na	na	na
0.91 (0.65, 1.42)	1.03 (0.62, 1.72)	Dapagliflozin	na	na
**0.74 (0.59, 0.92)**	0.84 (0.54, 1.30)	0.81 (0.54, 1.22)	Empagliflozin	na
0.95 (0.66, 1.39)	1.08 (0.64, 1.06)	1.04 (0.63, 1.73)	1.28 (0.84, 1.99)	Ertugliflozin

B. MACE

Pooled	**0.91 (0.85, 0.97)**			
Placebo	**0.86 (0.75, 0.97)**	0.93 (0.84, 1.03)	**0.86 (0.74, 0.99)**	0.97 (0.85, 1.11)
0.84 (0.70, 1.01)	Canagliflozin	na	na	na
0.92 (0.80, 1.07)	1.09 (0.87, 1.39)	Dapagliflozin	na	na
**0.84 (0.73, 0.96)**	0.99 (0.79, 1.25)	0.90 (0.74, 1.11)	Empagliflozin	na
0.97 (0.85, 1.11)	1.15 (0.91, 1.43)	1.04 (0.86, 1.27)	1.16 (0.96, 1.40)	Ertugliflozin

C. Hospitalization for heart failure

Pooled	**0.70 (0.62, 0.78)**			
Placebo	**0.67 (0.52, 0.87)**	**0.73 (0.61, 0.88)**	**0.65 (0.50, 0.85)**	**0.70 (0.54, 0.90)**
**0.62 (0.46, 0.84)**	Canagliflozin	na	na	na
**0.72 (0.61, 0.86)**	1.16 (0.82, 1.65)	Dapagliflozin	na	na
**0.63 (0.51, 0.79)**	1.02 (0.70, 1.49)	0.88 (0.66, 1.16)	Empagliflozin	na
**0.70 (0.57, 0.88)**	1.14 (0.78, 1.65)	0.98 (0.74, 1.28)	1.12 (0.82, 1.51)	Ertugliflozin

D. Composite Renal

Pooled	**0.61 (0.50, 0.74)**			
Placebo	**0.60 (0.47, 0.77)**	**0.53 (0.43, 0.66)**	**0.54 (0.40, 0.75)**	0.81 (0.63, 1.04)
**0.60 (0.44, 0.82)**	Canagliflozin	na	na	na
**0.52 (0.42, 0.64)**	0.87 (0.60, 1.26)	Dapagliflozin	na	na
**0.54 (0.40, 0.73)**	0.90 (0.59, 1.39)	1.04 (0.72, 1.49)	Empagliflozin	na
**0.79 (0.64, 0.95)**	1.31 (0.90, 1.88)	**1.49 (1.13, 1.99)**	**1.45 (1.01, 2.08)**	Ertugliflozin

Table S5. GRADE quality of evidence for the main outcomes.

Author(s):
Question: SGLT-2 inhibitors compared to Placebo for different selected outcomes
Setting:
Bibliography:

Certainty assessment							№ of patients		Effect		Certainty	Importance
№ of studies	Study design	Risk of bias	Inconsistency	Indirectness	Imprecision	Other considerations	SGLT-2 inhibitors	Placebo	Relative (95% CI)	Absolute (95% CI)		
**All-cause Mortality**												
5	randomised trials	not serious	not serious	not serious	not serious	none	1548/26755 (5.8%)	1289/20204 (6.4%)	**HR 0.83** (0.74 to 0.92)	**11 fewer per 1000** (from 16 fewer to 5 fewer)	⨁⨁⨁⨁ HIGH	CRITICAL
**MACE**												
5	randomised trials	not serious	not serious	not serious	not serious	none	2538/26755 (9.5%)	1963/20204 (9.7%)	**HR 0.89** (0.84 to 0.94)	**10 fewer per 1000** (from 15 fewer to 6 fewer)	⨁⨁⨁⨁ HIGH	CRITICAL
**Hospitalization for heart failure**												
5	randomised trials	not serious	not serious	not serious	not serious	none	642/26755 (2.4%)	712/20204 (3.5%)	**HR 0.83** (0.74 to 0.92)	**6 fewer per 1000** (from 9 fewer to 3 fewer)	⨁⨁⨁⨁ HIGH	IMPORTANT
**Composite Renal**												
5	randomised trials	not serious	not serious	not serious	not serious	none	704/26755 (2.6%)	851/20204 (4.2%)	**HR 0.68** (0.61 to 0.74)	**13 fewer per 1000** (from 16 fewer to 11 fewer)	⨁⨁⨁⨁ HIGH	IMPORTANT

CI: Confidence interval; **HR:** Hazard Ratio

Figure S1. Assessment of potential publication bias by outcome.

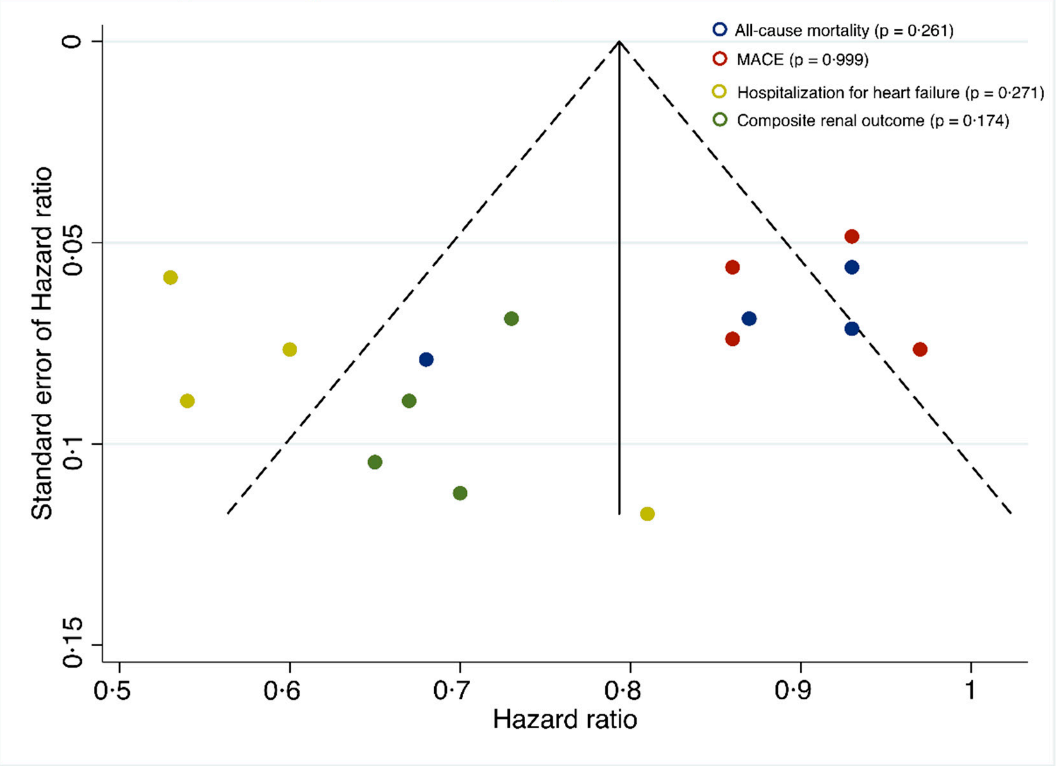

Table S6. PRISMA NMA Checklist of Items to Include When Reporting A Systematic Review Involving a Network Meta-analysis

Section/Topic	Item #	Checklist Item	Reported on Page #
**TITLE**			
Title	1	Identify the report as a systematic review *incorporating a network meta-analysis (or related form of meta-analysis).*	***1***
**ABSTRACT**			
Structured summary	2	Provide a structured summary including, as applicable:<br>**Background:** main objectives<br>**Methods:** data sources; study eligibility criteria, participants, and interventions; study appraisal; and *synthesis methods, such as network meta-analysis.*<br>**Results:** number of studies and participants identified; summary estimates with corresponding confidence/credible intervals; *treatment rankings may also be discussed. Authors may choose to summarize pairwise comparisons against a chosen treatment included in their analyses for brevity.*<br>**Discussion/Conclusions:** limitations; conclusions and implications of findings.<br>**Other:** primary source of funding; systematic review registration number with registry name.	2-3
**INTRODUCTION**			
Rationale	3	Describe the rationale for the review in the context of what is already known*, including mention of why a network meta-analysis has been conducted.*	***5-6***
Objectives	4	Provide an explicit statement of questions being addressed, with reference to participants, interventions, comparisons, outcomes, and study design (PICOS).	6
**METHODS**			
Protocol and registration	5	Indicate whether a review protocol exists and if and where it can be accessed (e.g., Web address); and, if available, provide registration information, including registration number.	6
Eligibility criteria	6	Specify study characteristics (e.g., PICOS, length of follow-up) and report characteristics (e.g., years considered, language, publication status) used as criteria for eligibility, giving rationale. *Clearly describe eligible treatments included in the treatment network, and note whether any have been clustered or merged into the same node (with justification).*	***6-7***
Information sources	7	Describe all information sources (e.g., databases with dates of coverage, contact with study authors to identify additional studies) in the search and date last searched.	6
Search	8	Present full electronic search strategy for at least one database, including any limits used, such that it could be repeated.	Sup. Table 1
Study selection	9	State the process for selecting studies (i.e., screening, eligibility, included in systematic review, and, if applicable, included in the meta-analysis).	6-7
Data collection process	10	Describe method of data extraction from reports (e.g., piloted forms, independently, in duplicate) and any processes for obtaining and confirming data from investigators.	7
Data items	11	List and define all variables for which data were sought (e.g., PICOS, funding sources) and any assumptions and simplifications made.	6-7
**Geometry of the network**	**S1**	Describe methods used to explore the geometry of the treatment network under study and potential biases related to it. This should include how the evidence base has been graphically summarized for presentation, and what characteristics were compiled and used to describe the evidence base to readers.	***9***
Risk of bias within individual studies	12	Describe methods used for assessing risk of bias of individual studies (including specification of whether this was done at the study or outcome level), and how this information is to be used in any data synthesis.	10
Summary measures	13	State the principal summary measures (e.g., risk ratio, difference in means). *Also describe the use of additional summary measures assessed, such as treatment rankings and surface under the cumulative ranking curve (SUCRA) values, as well as modified approaches used to present summary findings from meta-analyses.*	10-12
Planned methods of analysis	14	Describe the methods of handling data and combining results of studies for each network meta-analysis. This should include, but not be limited to:<br>• *Handling of multi-arm trials;*<br>• *Selection of variance structure;*<br>• *Selection of prior distributions in Bayesian analyses; and*	7-8

		• *Assessment of model fit.*	
**Assessment of Inconsistency**	**S2**	Describe the statistical methods used to evaluate the agreement of direct and indirect evidence in the treatment network(s) studied. Describe efforts taken to address its presence when found.	8
Risk of bias across studies	15	Specify any assessment of risk of bias that may affect the cumulative evidence (e.g., publication bias, selective reporting within studies).	**8**
Additional analyses	16	Describe methods of additional analyses if done, indicating which were pre-specified. This may include, but not be limited to, the following: • Sensitivity or subgroup analyses; • Meta-regression analyses; • *Alternative formulations of the treatment network; and* • *Use of alternative prior distributions for Bayesian analyses (if applicable).*	***8-9***
**RESULTS†**			
Study selection	17	Give numbers of studies screened, assessed for eligibility, and included in the review, with reasons for exclusions at each stage, ideally with a flow diagram.	Sup. Figure 1
**Presentation of network structure**	**S3**	Provide a network graph of the included studies to enable visualization of the geometry of the treatment network.	***NA***
**Summary of network geometry**	**S4**	Provide a brief overview of characteristics of the treatment network. This may include commentary on the abundance of trials and randomized patients for the different interventions and pairwise comparisons in the network, gaps of evidence in the treatment network, and potential biases reflected by the network structure.	***NA***
Study characteristics	18	For each study, present characteristics for which data were extracted (e.g., study size, PICOS, follow-up period) and provide the citations.	Table 1 and page 10
Risk of bias within studies	19	Present data on risk of bias of each study and, if available, any outcome level assessment.	12
Results of individual studies	20	For all outcomes considered (benefits or harms), present, for each study: 1) simple summary data for each intervention group, and 2) effect estimates and confidence intervals. *Modified approaches may be needed to deal with information from larger networks.*	***Table 1, 10-12***
Synthesis of results	21	Present results of each meta-analysis done, including confidence/credible intervals. *In larger networks, authors may focus on comparisons versus a particular comparator (e.g. placebo or standard care), with full findings presented in an appendix. League tables and forest plots may be considered to summarize pairwise comparisons.* If additional summary measures were explored (such as treatment rankings), these should also be presented.	***10-12, Figures 1-4***
**Exploration for inconsistency**	**S5**	Describe results from investigations of inconsistency. This may include such information as measures of model fit to compare consistency and inconsistency models, *P* values from statistical tests, or summary of inconsistency estimates from different parts of the treatment network.	***12***
Risk of bias across studies	22	Present results of any assessment of risk of bias across studies for the evidence base being studied.	12
Results of additional analyses	23	Give results of additional analyses, if done (e.g., sensitivity or subgroup analyses, meta-regression analyses*, alternative network geometries studied, alternative choice of prior distributions for Bayesian analyses,* and so forth).	***10-12***
**DISCUSSION**			
Summary of evidence	24	Summarize the main findings, including the strength of evidence for each main outcome; consider their relevance to key groups (e.g., healthcare providers, users, and policy-makers).	12-13
Limitations	25	Discuss limitations at study and outcome level (e.g., risk of bias), and at review level (e.g., incomplete retrieval of identified research, reporting bias). *Comment on the validity of the assumptions, such as transitivity and consistency. Comment on any concerns regarding network geometry (e.g., avoidance of certain comparisons).*	14-15
Conclusions	26	Provide a general interpretation of the results in the context of other evidence, and implications for future research.	15-16
**FUNDING**			
Funding	27	Describe sources of funding for the systematic review and other support (e.g., supply of data); role of funders for the systematic review. This should also include information regarding whether funding has been received from manufacturers of treatments in the network and/or whether some of the authors are content experts with professional conflicts of interest that could affect use of treatments in the network.	***10***
